# A longitudinal evaluation of improvements in treatment plan quality for lung cancer with volumetric modulated arc therapy

**DOI:** 10.1002/acm2.12863

**Published:** 2020-04-01

**Authors:** Wenlong Xia, Zhiqiang Liu, Lingling Yan, Fei Han, Zhihui Hu, Yuan Tian, Weijie Cui, Wenting Ren, Chenlei Guo, Junjie Miao, Jianrong Dai

**Affiliations:** ^1^ Department of Radiation Oncology National Cancer Center/National Clinical Research Center for Cancer/Cancer Hospital Chinese Academy of Medical Sciences and Peking Union Medical College Beijing China

**Keywords:** lung cancer, OAR sparing, plan quality, planning time, VMAT

## Abstract

**Purpose:**

To investigate planning time and number of optimizations in routine clinical lung cancer plans based on the plan quality improvements following each optimization.

**Materials and method:**

We selected 40 patients with lung cancer who were treated with conventional fractionated radiotherapy (CFRT). The 40 plans (divided into two groups with one or two target volumes) were completed by 9 planners using volumetric modulated arc therapy (VMAT). A planning strategy, including technique script for each group and a planning process for data collection, was introduced. The total planning time, number of optimizations, and dose–volume parameters of each plan were recorded and analyzed. A plan quality metric (PQM) was defined according to the clinical constraints. Statistical analysis of parameters of each plan following each optimization was performed for evaluating improvements in plan quality.

**Results:**

According to the clinical plans generated by different planners, the median number of optimizations of each group was 4, and the median planning time was approximately 1 h (68.6 min and 62.0 min for plans with one or two target volumes, respectively). The dose deposited in organs at risk (OARs) gradually decreased, and the PQM values gradually improved following each optimization. The improvements were significant only between adjacent optimizations from the first optimization (Opt1) to the third optimization (Opt3).

**Conclusion:**

Increasing the number of optimizations was associated with significantly improved sparing of OARs with slight effects on the dose coverage and homogeneity of target volume. Generally, based on the designed planning strategy, there was no significant improvement of the plan quality for more than three optimizations.

## 1 INTRODUCTION

Radiotherapy is a major treatment modality for lung cancer. Several studies show the potential of volumetric modulated arc therapy (VMAT) compared with that of intensity‐modulated radiotherapy (IMRT) for reducing treatment time without compromising the plan quality.^1^, ^2^, ^3^ In practice, the optimization of VMAT depends on the parameters involving the number of arcs, delivery time, collimator angles, field size, or the gantry angle spacing between subsequent control points. These parameters are often selected manually via trial‐and‐error according to the planner’s experience.

Due to the complexity of treatment plans, the planning process is usually iterative and time consuming. Planning time of the standard planning process is dominated by performing optimization iterations with the system (i.e., setting parameters, performing the optimization, evaluating the results, and repeating these steps until the planners are satisfied).^4^ The planning time is an important factor that can be used as not only a significant reference for both planners and physicians but also an important data for the administrator of the department to improve the management of clinical workflow. The investigation of average planning time is mainly based on the statistical data of large number of clinical plans. Therefore, we employed 40 lung cancer plans and designed a planning strategy to obtain time consumption of each plan.

The quality of treatment plans would also vary considerably among different planners and institutions,^5^ which means sub‐optimal treatment plans may be produced. During plan optimization process, two situations may occur. One involves insufficient optimization, although the clinical requirements are achieved, the dose distribution can be improved through further optimization. The other situation involves over‐optimization, in which the plan is optimized beyond a certain number of optimizations without significantly improving plan quality. To investigate the gains in plan quality improvement during optimization, we performed an analysis of longitudinal dosimetric trends by comparing adjacent optimizations in the planning process.

To conduct a comprehensive assessment of a treatment plan, it is necessary to compare each metric of the target volumes and the OARs, as well as the overall plan quality according to a quantitative evaluation criterion. Certain quantitative evaluation methods are mainly used to compare the quality of the same plan finished by different planners, different institutions, different TPSs, or different modalities. These methods cannot directly be used to evaluate statistical control experiments. Thus, according to the existing plan quality metric (PQM)^5^ and quality score S_D_,^6^ here we introduce a new plan quality scoring procedure for lung cancer.

This study comprised two parts. The first involved the statistics of planning time and number of optimizations of the resultant lung cancer plans. According to the designed planning strategy, 40 plans were completed by nine planners with different years of experiences, and corresponding data were recorded. The second involved treatment planning improvement following each optimization of a plan. An analysis of longitudinal dosimetric changes was performed, and the new PQM scoring procedure was used to quantify treatment plan quality.

## 2 MATERIALS AND METHODS

### 2.A Patient selection and planning objectives

To include homogenous patient population, treatment plans of 40 patients with lung cancer who underwent conventional fractionated radiotherapy (CFRT) were selected from recent clinical treatment plans. The patients were scanned during normal breathing in the supine position using 5 mm slice thickness computed tomography (CT) in plane voxel size of 1 mm × 1 mm. Gross tumor volume (GTV), planning gross tumor volume (PGTV), clinical target volume (CTV), and planning target volume (PTV) were contoured by qualified radiation oncologists. Other relevant OARs were delineated, which mainly included the whole lung, spinal cord, and heart. A 5 mm margin was added to the spinal cord as the planning organ at risk volume (PRV).

Twenty plans included one target volume (single PTV), the mean tumor size was 387.4 ± 180.6 cm^3^ (36.3–754.8 cm^3^; median, 365.0 cm^3^). The others included PGTV and PTV, the PGTV was subjected to local dose escalation, while the prescribed dose was simultaneously delivered to the PTV, the so called “simultaneous integrated boost.” The mean tumor size of the PGTV was 162.2 ± 108.6 cm^3^ (19.8–353.3 cm^3^, median 149.2 cm^3^), and the mean tumor size of the PTV was 377.4 ± 164.1 cm^3^ (60.0–706.4 cm^3^, median 396.7 cm^3^). Patients’ characteristics are summarized in Table 1. The location of each tumor was defined as in the right or left lung depending on the location of the primary lesion and superior or inferior depending on whether >50% of the PTV was located superior or inferior to a line bisecting the lungs.

**Table 1:** Patient characteristics.

	Number of patients
Stage	
Single PTV	
T4	6(N2 = 3/N3 = 3)
T3	3(N0 = 2/N2 = 1)
T2	6(N1 = 1/N2 = 3/N3 = 2)
T1	5(N0 = 1/N2 = 3/N3 = 1)
PGTV and PTV	
T4	4(N2 = 1/N3 = 3)
T3	8(N1 = 3/N2 = 3/N3 = 2)
T2	5(N1 = 1/N2 = 2/N3 = 2)
T1	3(N0 = 1/N3 = 2)
Location	
Single PTV	
Right	9
Left	11
Upper	15
Lower	5
PGTV and PTV	
Right	15
Left	5
Upper	17
Lower	3
Prescribed dose/fractionation	
Single PTV	
60 Gy/30 × 2 Gy	20
PGTV and PTV	
60.2 Gy/28 × 2.15 Gy;50.4 Gy/28 × 1.8 Gy	6
59.92 Gy/28 × 2.14 Gy;50.4 Gy/28 × 1.8 Gy	4
63 Gy/30 × 2.1Gy;54 Gy/30 × 1.8 Gy	4
60 Gy/30 × 2 Gy;54 Gy/30 × 1.8 Gy	3
58.8 Gy/28 × 2.1 Gy;50.4 Gy/28 × 1.8 Gy	2
61.88 Gy/28 × 2.21 Gy;50.4 Gy/28 × 1.8 Gy	1

Abbreviations: PGTV, planning gross tumor volume; PTV, planning target volume.

The planning objectives for PTVs were as follows: the relative volume that receives 100% of the prescribed dose >95%; and maximum point dose <110% of prescribed dose. The dose coverage and homogeneity of PTVs were assessed using dose distribution and dose–volume histogram (DVH), as well as considering the trade‐off of dose delivered to PTV and OAR sparing. Other planning objectives for the OARs were as follows: point dose, spinal cord < 40 Gy; point dose, spinal cord PRV < 45 Gy; volume of whole lung receiving more than 5 Gy (V5) is not specified (the lower the better), and more than 20 Gy (V20) < 28%; mean dose, whole lung *D*_mean_ < 17 Gy; volume of heart receiving more than 30 Gy (V30) < 40% and more than 40 Gy (V40) < 30%. The 40 plans were finished by nine planners with 3–10 yr working experiences.

### 2.B Design of planning strategy

VMAT treatment plans were optimized using the SmartArc optimization engine implemented in Pinnacle^3^ v9.10 (Philips Medical System, Fitchburg, WI). SmartArc is an extension of the Direct Machine Parameter Optimization (DMPO) planning functionality in Pinnacle. The integrated SmartArc module uses a dynamic arc optimization process developed for VMAT and intensity‐modulated arc therapy (IMAT).^7^, ^8^, ^9^ The dose calculating model employed the Collapsed Cone Convolution (CCC) algorithm, 4 mm dose grid resolution. CCC algorithm is a three‐dimensional dose computation that intrinsically handles the effects of the patient heterogeneities on both primary and secondary scattered radiation.^10^

A planning strategy was designed for the 40 plans, which mainly include two parts: (a) optimization technique script created for each group; (b) planning process designed for obtaining the required information.

The optimization technique script in Pinnacle was commonly used for standardizing the first optimization and improving planning efficiency. Before running the script, several operations were completed, which included defining the CT‐density table, creating an isocenter of the beams, removing the CT couch, and contouring the outline of the body. The description of optimization script was as follows: First, the required dose‐shaping structures (DSS) including rings of the targets, normal tissue (NT), blocks, the fan areas above and below the targets (Fan up and Fan down) were created according to relevant Boolean operations. Next, clockwise (CW) and counterclockwise (CCW) arcs were created, ranging from 181º to 30º for tumors located in right lung and from 330º to 180º for tumors located in left lung. In the script, we used Varian Novalis Tx equipped with the 120 multileaf collimator (MLC) (Field size 40 × 40 cm, Central 20 cm of field – 5 mm leaf width, Outer 20 cm of field – 10 mm leaf width). Settings were as follows: beam energy 6 MV, control point spacing 4º, and the leaf motion constrained to 0.5 cm/deg. Finally, the initial settings of planning objectives were defined according to the clinical requirements.

**Fig. 1:**
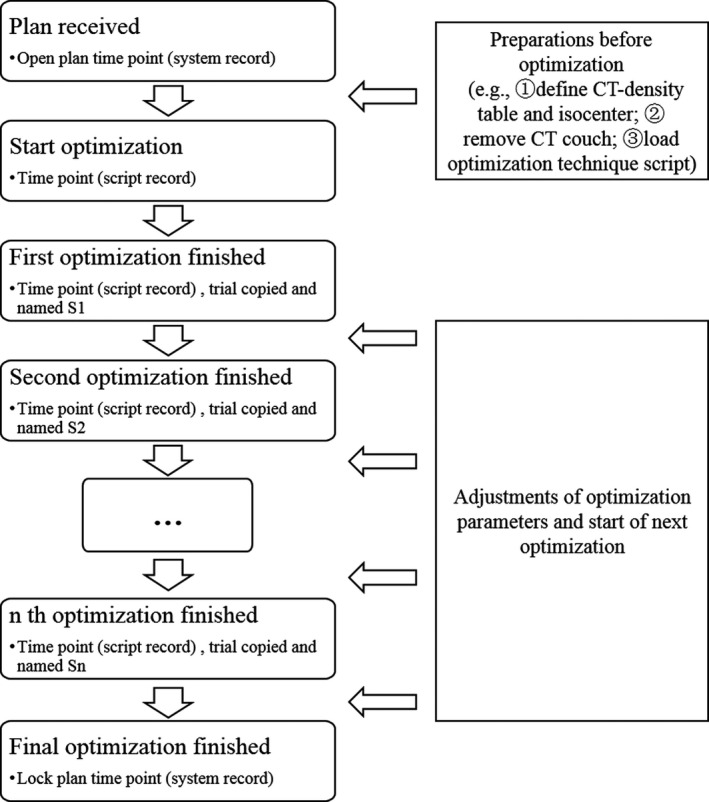
Planning process for standardizing the optimization and collecting plan information.

In the planning process, information including time points and evaluation parameters of the plans after each optimization was obtained (Fig. 1). First, when we received a plan, the system automatically recorded the plan open time. Next, the preparations (e.g., defining the CT‐density table, creating an isocenter of the beams, removing the CT couch, loading the optimization technique script) were completed before optimization. Finally, the optimization process was started using a computer script for recording the time, and plan following each optimization was manually copied for further analysis. The time points, which included the plan open time, start time and end time of each optimization, and the plan lock time, were recorded. Before starting the next optimization, necessary region of interest (ROI) can be created and each parameter was required to be set to more stringent conditions, meaning that each objective was tuned properly, and the optimization process continued with objective values >0. The 40 plans were optimized using the same hardware configuration. The collection of required information did not significantly interfere with the routine clinical planning design.

### 2.C Study endpoints

The 40 plans were divided into a group of plans with one target volume PTV and a group with two target volumes (PTV and PGTV). Different optimizations were compared using metrics averaged over the 40 plans of each optimization as follows:

- Homogeneity index (HI) is defined as:

(1) $$\text{HI}=100\%\times \frac{D2\%-D98\%}{D50\%}$$

Here D2%, D50%, and D98% are minimum doses delivered to 2%, 50%, and 98% of the PTV, respectively. The closer the value of HI is to 0, the better is the homogeneity of PTV.^11^

- Conformity index (CI) is defined as:

(2) $$\text{CI}=\left(TV_{\mathrm{PV}}/V_{\mathrm{PTV}}\right)/\left(V_{\mathrm{TV}}/TV_{\mathrm{PV}}\right)$$

V_PTV_ is the volume of PTV. TV_PV_ is the portion of the V_PTV_ within the prescribed isodose line. V_TV_ is the treated volume of the prescribed isodose line. The closer the value of CI is to 1, the better is the conformity of PTV.^12^

- Dose deposition in lungs was analyzed using V5 Gy (%), V20 Gy (%), and mean dose (D_mean_).
- Dose deposition in the heart was analyzed using V30 Gy (%), V40 Gy (%).
- Maximum dose (D_max_) to spinal cord and spinal cord PRV.

These metrics were determined according to the patients’ clinical requirements.

### 2.D Plan quality metrics

A new Plan Quality Metric (PQM) with related submetrics was defined for a lung cancer treatment plan. We employed 9 subcomponents for the first group of plans with a single target volume and 11 subcomponents for the second group of plans with 2 target volumes, each with unique metric quantity and PQM value functions that were used to calculate a point value according to the submetric (Table 2). For each metric in the first group of plans with a single target volume PTV, the ranges of the corresponding PQM values were uniformly set from 0 to 10. For the metrics of the second group of plans with the target volumes PTV and PGTV, the ranges of PQM values for CI (PTV), CI (PGTV), HI (PTV), and HI (PGTV) were set from 0 to 5. The quality score S of each plan is the sum of PQM values of the subcomponents,^5^, ^6^ defined as follows:

(3) $$S=\sum_{i=1}^{k}S_{i}$$

(4) $$S_{i}=\left\{\begin{array}{c} \frac{M_{i}-M_{\mathrm{il}}}{M_{\mathrm{iu}}-M_{\mathrm{il}}}\times PQMvalue_{\mathrm{imax}},CI\\ \frac{M_{\mathrm{iu}}-M_{i}}{M_{\mathrm{iu}}-M_{\mathrm{il}}}\times PQMvalue_{\mathrm{imax}},else \end{array}\right.$$

k is the number of subcomponents, *S*_i_ is PQM value of corresponding metric (*M*_i_), *M*_il_ and *M*_iu_ are the lower limit and upper limit of *M*_i_, respectively. PQMvalue_imax_ is the maximum value (highest score) of each metric. The interval of each metric was determined for all recorded data of the 40 plans. Thus, all lung cancer plans in this control experiment could be evaluated using this PQM scoring procedure.

**Table 2:** Evaluation interval of metric parameters along with their value range.

Structure	Metric				PQM value range	
	Parameter	lower limit	interval	upper limit	minimum	maximum
PTV(single)	CI	0.4	0.4–1	1	0	10
	HI	0	0–0.5	0.5	0	10
PTV and PGTV	CI (PTV)	0.4	0.4–1	1	0	5
	CI (PGTV)	0.4	0.4–1	1	0	5
	HI (PTV)	0	0‐0.5	0.5	0	5
	HI (PGTV)	0	0–0.5	0.5	0	5
Lung all	V_5_ (%)	20	20–70	70	0	10
Lung all	V_20_ (%)	0	0–28	28	0	10
Lung all	D_mean_ (Gy)	0	0–17	17	0	10
Heart	V_30_ (%)	0	0–40	40	0	10
Heart	V_40_ (%)	0	0–30	30	0	10
Cord	D_max_ (Gy)	0	0–40	40	0	10
Cord PRV	D_max_ (Gy)	0	0–45	45	0	10

Abbreviations: PGTV, planning gross tumor volume; PRV, planning organ at risk volume; PQM, plan quality metric; PTV, planning target volume.

### 2.F Statistical analysis

The dosimetric data are summarized per optimization using mean ± SD and confidence intervals. Statistical analysis was performed using SPSS v17 (IBM Corp). The paired t test was adopted to compare the intergroup difference of data, and *P* < 0.05 indicates a significant difference.

## 3 RESULTS

**Fig. 2:**
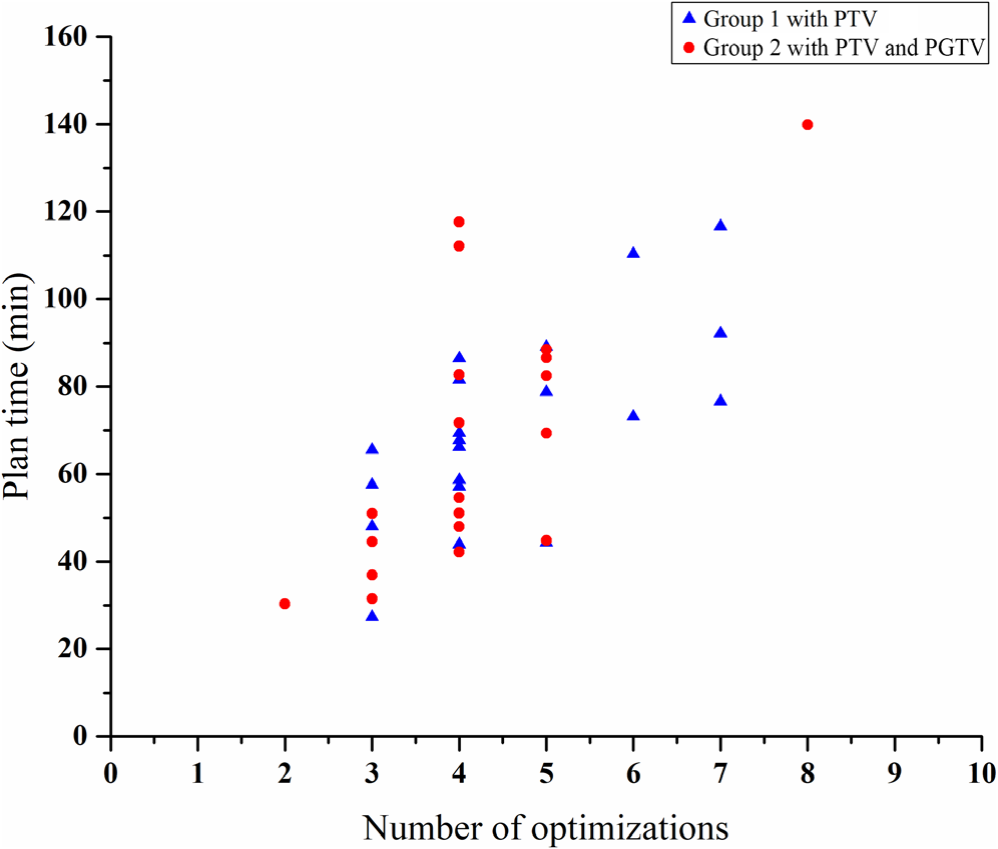
The relationship of planning time and number of optimizations.

According to the recorded information of each plan, the planning time and number of optimizations of the 40 plans was shown in Fig. 2. The planning time is the sum of the preparation time before optimization, parameter adjustment time before the next optimization, and the optimization time. The first two variables were mainly affected by the planners’ experience, and the third variable depended on the parameter settings and hardware condition of the treatment planning system and complexity of the plan. For the first group of plans with PTV, the average planning time was 70.5 ± 22.1 min, the median planning time was 68.6 min, and the corresponding median number of optimizations was 4. For the second group of plans with PTV and PGTV, the average planning time was 68.4 ± 30.4 min, the median planning time was 62.0 min, and the corresponding median number of optimizations was 4.

**Fig. 3:**
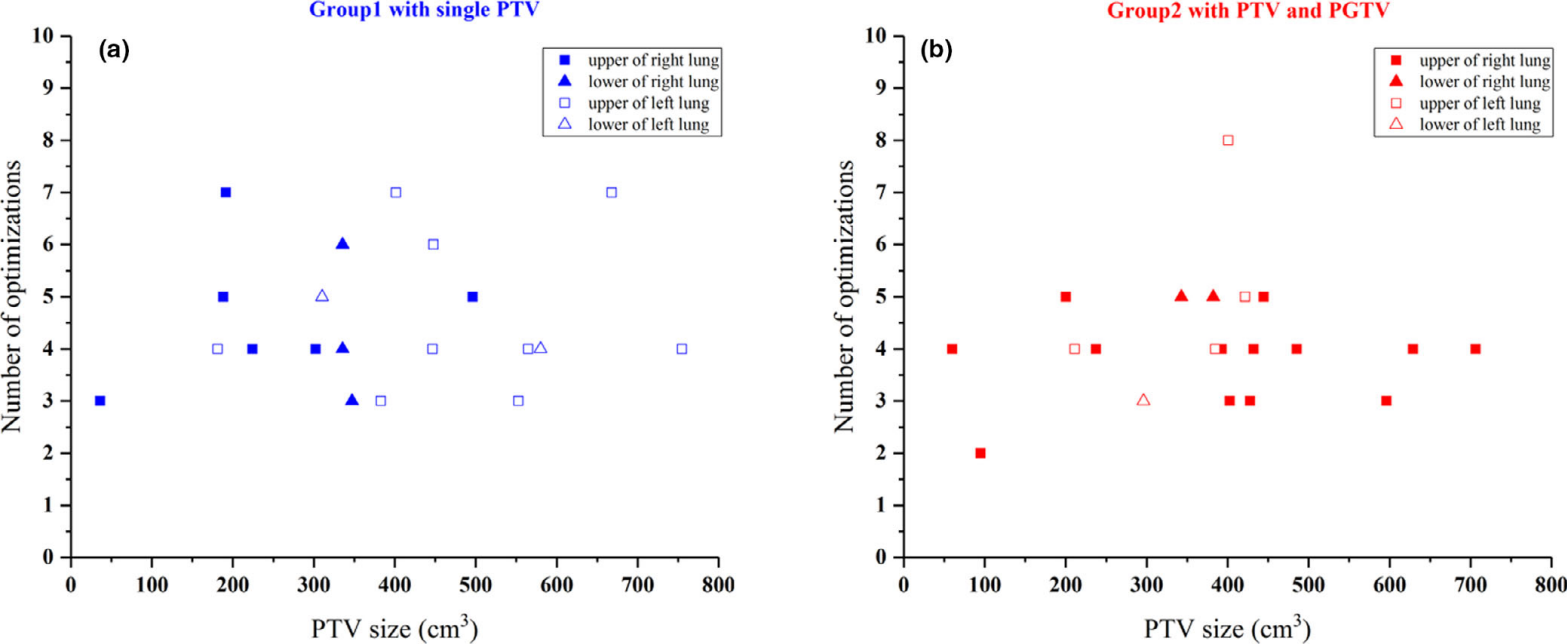
The relationship of number of optimizations and PTV size, PTV location. (a) Group1 with single PTV, (b) Group2 with PTV and PGTV. PGTV, planning gross tumor volume; PTV, planning target volume.

Figure 3 shows the relationship between the number of optimizations and tumor size. The tumor locations are marked in the figure. For both groups, there was no obvious consistency associated with the relationship between number of optimizations and tumor size, or between number of optimizations and tumor location.

**Fig. 4:**
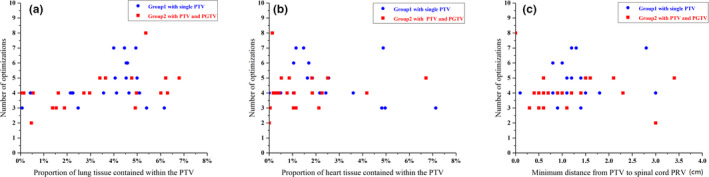
The relationship of number of optimizations and (a) proportion of lung tissue contained within the PTV, (b) proportion of heart tissue contained within the PTV, (c) the minimum distance from PTV to spinal cord PRV. PRV, planning organ at risk volume; PTV, planning target volume.

The relationships between the number of optimizations and variables related to OAR‐PTV distance are shown in Fig 4. The variables include the proportion of lung tissue within the PTV, the proportion of heart within the PTV, and the minimum distance from the PTV to the spinal cord PRV. There were no statistically significant consistencies associated with the relationships between the number of optimizations and any of the variables. Even so, it still can be observed that there was an increasing trend of the number of optimizations associated with the proportion of lung tissue within the PTV.

**Fig. 5:**
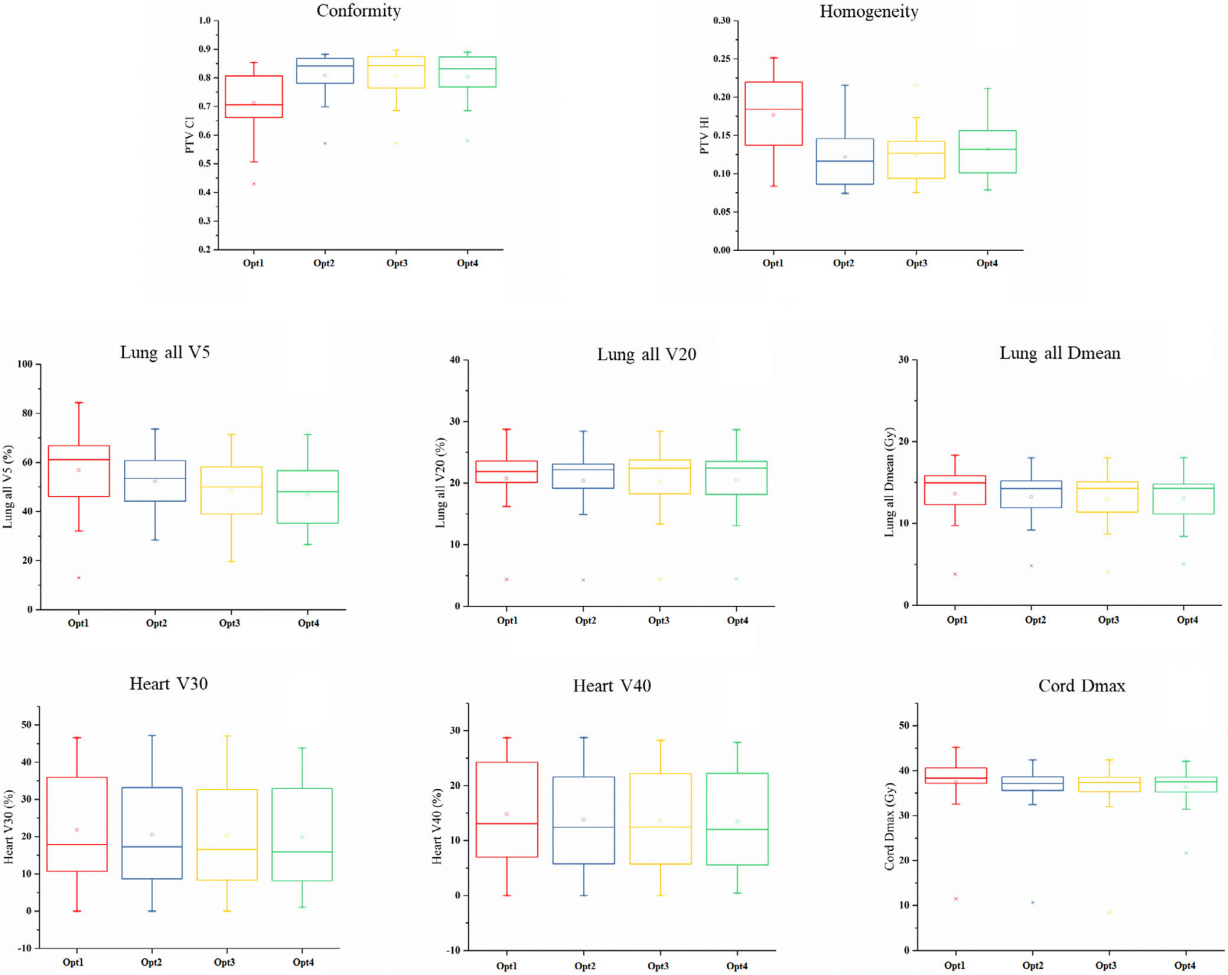
Box–whisker plots showing the spread of metrics for the plans with single PTV. PTV, planning target volume.

**Fig. 6:**
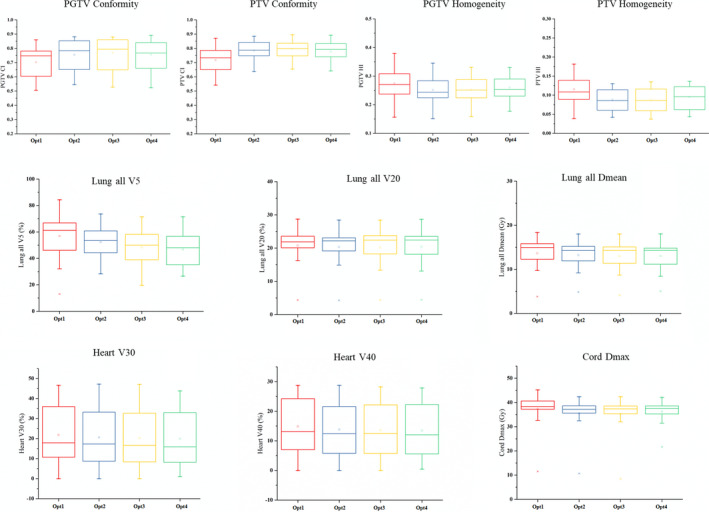
Box–whisker plots showing the spread of metrics for the plans with PTV and PGTV. PGTV, planning gross tumor volume; PTV, planning target volume.

The gradual changes in metrics from Opt1 to Opt4 are shown in Figs 5 and 6. There were similar trends in the distributions of metrics of the two groups. The CI and HI of target volumes exhibited an apparent improvement from Opt1 to Opt2 and were slightly worse from Opt2 to Opt4. The distributions of the metrics of lungs and heart gradually improved from Opt1 to Opt4, while the *D*_max_ (Gy) of the spinal cord slightly changed during optimization.

**Fig. 7:**
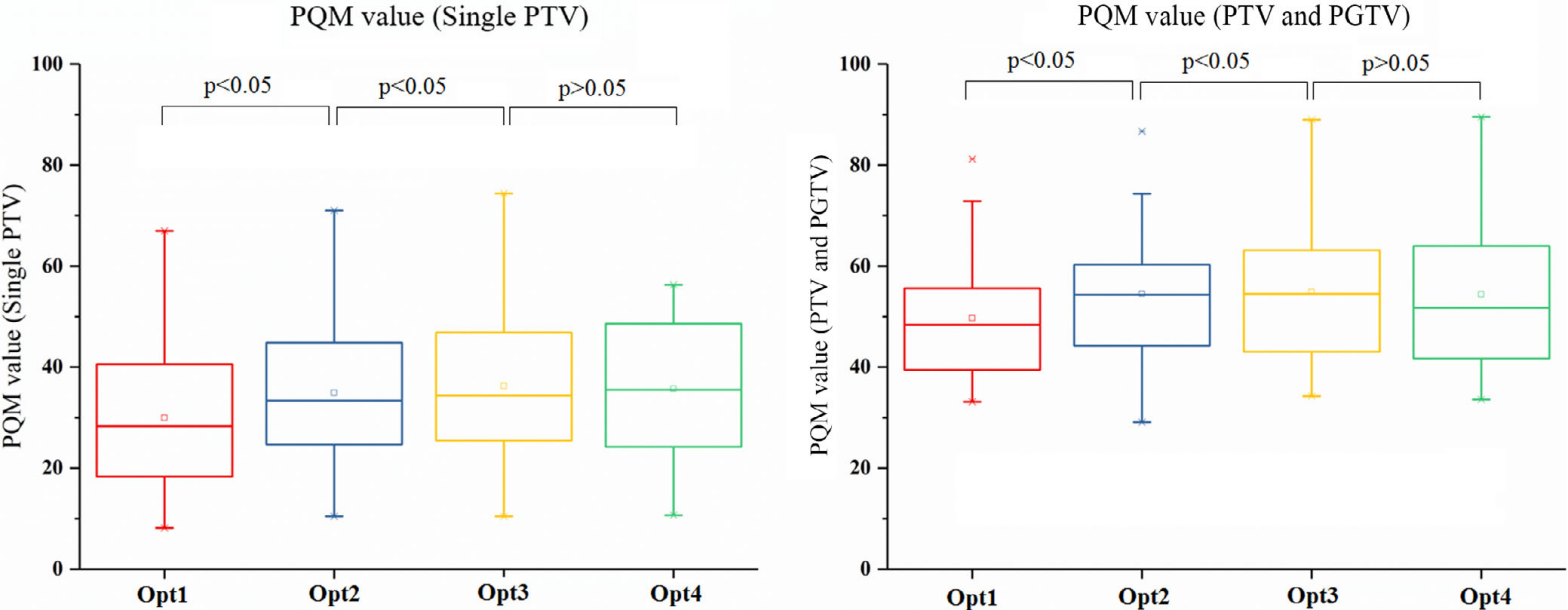
Box–whisker plots showing the spread of PQM values. PQM, plan quality metric.

Figure 7 shows the changes in PQM values of the two groups, indicating plan quality improved following the optimization process. As the number of optimizations increased, the range of improvement tended not to change.

Tables 3 and 4 show the averaged differences (Diff) of the metrics between the adjacent optimizations from Opt1 to Opt4 in the two groups, respectively. The Diff of each metric was defined as the average value of the differences between Opt n and Opt n + 1 (value of Opt n − value of Opt n + 1, where n is 1 to 3) for all 20 cases in each group.

**Table 3:** Averaged differences and 95% confidence interval between the adjacent optimizations in the first group of plans with single PTV.

Phase	Opt1 vs Opt2		Opt2 vs Opt3		Opt3 vs Opt4	
	Diff. (95% confidence interval)	*P*	Diff. (95% confidence interval)	*P*	Diff. (95% confidence interval)	*P*
PTV (single)						
CI (PTV)	−0.096 (−0.127 to −0.066)	0.000	0.000 (−0.008 to 0.007)	0.977	0.011 (−0.000 to 0.022)	0.053
HI (PTV)	0.055 (0.040 to 0.069)	0.001	−0.002 (−0.008 to 0.005)	0.594	−0.007 (−0.011 to −0.003)	0.001
Lung all						
V5 (%)	4.56 (1.51 to 7.61)	0.006	3.94 (2.40 to 5.47)	0.000	2.40 (1.27 to 3.52)	0.000
V20 (%)	0.40 (0.10 to 0.70)	0.012	0.20 (−0.15 to 0.56)	0.245	0.10 (−0.04 to 0.24)	0.156
Dmean (Gy)	0.39 (0.18 to 0.61)	0.001	0.26 (0.06 to 0.29)	0.003	0.18 (0.06 to 0.29)	0.006
Heart						
V30 (%)	1.19 (0.48 to 1.91)	0.002	0.39 (0.00 to 0.78)	0.048	0.01 (−0.30 to 0.32)	0.936
V40 (%)	1.02 (0.41 to 1.64)	0.002	0.25 (−0.21 to 0.70)	0.268	−0.09 (−0.30 to 0.12)	0.354
Spinal cord						
Dmax (Gy)	1.88 (1.10 to 2.65)	0.000	0.28 (−0.38 to 0.94)	0.390	−0.03 (−0.59 to 0.53)	0.904
Spinal cord PRV						
Dmax (Gy)	1.51 (0.36 to 2.66)	0.013	0.73 (0.08 to 1.38)	0.030	0.12 (−0.72 to 0.96)	0.766
Treatment plan quality assessment						
PQM value	−4.92 (−5.91 to −3.93)	0.000	−1.37 (−1.91 to −0.82)	0.000	−0.33 (−0.92 to −0.26)	0.253

Abbreviations: PRV, planning organ at risk volume; PQM, plan quality metric; PTV, planning target volume.

**Table 4:** Averaged differences and 95% confidence interval between the adjacent optimizations in the second group of plans with PTV and PGTV.

Phase	Opt1 vs Opt2		Opt2 vs Opt3		Opt3 vs Opt4	
	Diff. (95% confidence interval)	*P*	Diff. (95% confidence interval)	*P*	Diff. (95% confidence interval)	*P*
PGTV						
CI (PGTV)	−0.053 (−0.079 to −0.027)	0.000	−0.009 (−0.021 to 0.004)	0.154	0.004 (−0.007 to 0.015)	0.464
HI (PGTV)	0.023 (−0.125 to 0.000)	0.049	−0.011 (−0.048 to 0.025)	0.496	0.016 (−0.006 to 0.038)	0.132
PTV						
CI (PTV)	−0.068 (−0.102 to −0.033)	0.001	0.010 (−0.028 to 0.008)	0.281	0.010 (−0.005 to 0.025)	0.169
HI (PTV)	0.027 (0.016 to 0.039)	0.000	0.001 (−0.003 to 0.005)	0.539	−0.005 (−0.009 to −0.001)	0.015
Lung all						
V5 (%)	2.44 (1.49 to 3.40)	0.000	1.56 (0.75 to 2.36)	0.001	0.97 (−0.06 to 2.01)	0.063
V20 (%)	0.75 (0.19 to 1.31)	0.011	0.39 (0.11 to 0.66)	0.009	0.37 (−0.09 to 0.83)	0.108
Dmean (Gy)	0.39 (0.22 to 0.56)	0.000	0.27 (0.17 to 0.36)	0.001	0.11 (−0.02 to 0.25)	0.100
Heart						
V30 (%)	0.62 (−0.09 to 1.34)	0.083	0.49 (0.18 to 0.79)	0.003	0.23 (−0.37 to 0.84)	0.422
V40 (%)	0.71 (0.17 to 1.26)	0.013	0.16 (−0.07 to 0.40)	0.166	−0.03 (−0.32 to 0.25)	0.799
Spinal cord						
Dmax (Gy)	1.18 (0.56 to 1.80)	0.001	0.61 (0.10 to 1.12)	0.022	0.20 (−0.29 to 0.69)	0.400
Spinal cord PRV						
Dmax (Gy)	0.82 (−0.20 to 1.84)	0.109	0.51 (−0.02 to 1.04)	0.057	0.91 (−0.56 to 2.38)	0.207
Treatment plan quality assessment						
PQM value	−4.86 (−6.74 to −2.98)	0.000	−1.33 (−2.16 to −0.50)	0.003	−0.21 (−0.80 to 0.37)	0.439

Abbreviations: PGTV, planning gross tumor volume; PRV, planning organ at risk volume; PQM, plan quality metric; PTV, planning target volume.

For the first group of plans with single target volume, the CI and HI were improved after the second optimization (Opt2), and the HI was significantly worse after the fourth optimization (Opt4) (*P* < 0.05). The metrics of both lungs were significantly different, except for those of V20 from Opt2 to Opt4. The other metrics including V5, V20, and D_mean_ gradually and significantly (*P* < 0.05) decreased from Opt1 to Opt4. For the metrics of heart, V30 and V40 were slightly decreased from Opt1 to Opt4, and the differences of V30 from Opt1 to Opt3 and V40 between Opt1 and Opt2 were significant (*P* < 0.05). The maximum dose delivered to spinal cord was slightly reduced after Opt2, and the maximum dose delivered to spinal cord PRV gradually and significantly (*P* < 0.05) decreased from Opt1 to Opt3.

The results for the second group of plans with two target volumes were similar to those of the first group of plans with single target volume. The CI and HI improved after Opt2 for PTV and PGTV, and the HI of PGTV was significantly (*P* < 0.05) worse after Opt4. The V5, V20, and D_mean_ of lungs gradually and significantly (*P* < 0.05) decreased from Opt1 to Opt3. For the metrics of heart, the differences of V30 between Opt2 and Opt3, as well as V40 between Opt1 and Opt2 were significant (*P* < 0.05). The maximum dose to the spinal cord was significantly (*P* < 0.05) reduced from Opt1 to Opt3.

For both groups, the improved OAR sparing from Opt1 to Opt4 did not have a significant effect on the CI and HI of the target volumes.

## 4 DISCUSSION

Here we conducted a statistical analysis on planning time and number of optimizations through improvements along successive optimizations to evaluate average performances in the routine clinical lung cancer treatment plans.

The optimization technique script and planning process developed here were used as the planning strategy for lung cancer patients who were treated with CFRT. Generally, radiotherapy treatment planning involves two basic steps as follows: (a) preparation before optimization, including the delineation of auxiliary anatomy, arrangement of beam angles, and initial optimization of parameter settings; (b) optimization process including modification of a set of dose–volume points and weights until achieving the development of a plan that satisfied the planners. For the cases with the same clinical requirements and similar features of target volumes (e.g., size and prescription), the first step can be completed by running the same technique script, which was developed according to years’ planning experience inhouse. The planning strategy used here efficiently standardized the preparations in the first step while focusing on the optimization process in the second step. Recently, an optimization workflow using design of experiment (DoE) tool for various field configurations was developed by Miki.^13^ This workflow aims at decreasing trial‐and‐error of field arrangement parameters and providing personalized objective suggestions associated with only the geometrical relationships between the target and the OARs. The experience‐based technique script in this study and personalized DoE tool can efficiently standardize and improve treatment plan quality. Such approaches can be applied to auto‐planning or knowledge‐based planning,^14^, ^15^ in which field configurations and initial optimization parameters still need to be set manually.

The statistical results showed that the planning time increases along with the number of optimizations, and there was increasing trend of the number of optimizations associated with the proportion of lung tissue within the PTV. However, there was no significant consistency for the relationship between the number of optimizations and other variables. The results indicate that the increasing proportion of lung tissue in the PTV raises the difficulty of the lung cancer VMAT plan, and planners are more concerned with lung tissue sparing when the heart and spinal cord have met the clinical goals. The average planning time was 70.5 ± 22.1 min (median 68.6 min) for plans with a single PTV in the first group and 68.4 ± 30.4 min (median 62.0 min) for plans with PTV and PGTV in the second group. The target of the second group was more complex. However, the average planning time of the second group was slightly shorter. This could be attributed to the difference in prescriptions between the two groups. For the first group, the prescribed dose of PTV was 60 Gy. However, in the second group, the prescribed dose of PTV was approximately 50 Gy, while the prescribed dose of PGTV was approximately 60 Gy. The lower dose of PTV in the second group might reduce the difficulty of the plan. The statistical results of planning time will help both planners and physicians aware of average time consumption of routine clinical lung cancer plans. The method can also be used to other tumor sites, so that the administrator can further improve the management of clinical workflow refer to these statistical results.

According to the results of our present statistical analysis on the plan quality improvements from Opt1 to Opt4 in the two groups of plans, we found that advances were made throughout the optimizations that were significantly associated with dose reduction delivered to OARs. The gradual sparing of the OARs had only a slight effect on CI and HI of target volumes. The average differences from Opt1 to Opt4 were significantly associated with improved dose deposition in lung tissue. In contrast, there were no obvious improvements in the dose distributions to heart and spinal cord when the metrics met the clinical goals. In this study, paired t tests were performed between the adjacent optimization from Opt1 to Opt4, and there were significant improvements of plan quality found from Opt1 to Opt3. Since no significant improvement was found between Opt3 and Opt4, the analysis on subsequent optimizations (from Opt5 to Opt8) was not involved. There was no significant improvement of lung cancer plans for more than three optimizations. This result could be especially valuable to the planners, so that they will have a general grasp of proper number of optimizations in planning process of this type of lung cancer plans.

The PQM proposed by Benjamin^5^ and the plan quality score S_D_ proposed by Bohsung^6^ were mainly used to compare the quality of the same plan (one case) finished by different planners, different institutions, different TPSs, or different modalities. These methods cannot directly be used to evaluate statistical control experiments of different plans. On the basis of PQM and S_D_, the new PQM scoring procedure used different definition of evaluated interval. The upper and lower limits of each metric were defined by clinical requirements as well as the range of the collected data of all 40 plans. Thus, different plans (all 40 cases) can use the same PQM scoring procedure. The evaluated metrics were the standard clinical OARs’ constraints to lung cancer, and the quality score assignments of different metrics were determined by both physicians and planners, who are experts in lung cancer radiotherapy. The PQM scoring procedure should be individually developed according to different tumor sites. For example, the radiotherapy plans for nasopharyngeal cancer require better dose coverage and homogeneity of target volumes compared with those for lung cancer plans, and the proportions of scores of target volumes should be increased.

As described above, here we conclude the main outputs of this study: (a) Present a planning strategy for standardizing the optimization and collecting the statistical data; (b) Statistical analyses of planning time and the number of optimizations in routine clinical planning process; (c) Obtain average boundary for lung cancer via longitudinal approach based on a large number of clinical plans; (d) Introduce a new PQM scoring procedure for evaluating the quality of a treatment plan. However, limitations are still existed in this study: (a) For a specific plan, both planning time and number of optimizations are dependent on various factors, such as treatment technique, planning strategy (initial settings of parameters), optimization engine, dose calculation algorithm, planner's experience, complexity of the plan (tumor size and location, and factors related to PTV‐OAR distance, etc). Although the designed planning strategy was used and the statistical data of one type of plan could be generally referred, the actual planning time and number of optimizations of a plan still depend on some factors (especially for initial settings and skill of planner). (b) The nine planners belong to same institute, and different institutes may use different planning strategy, which may lead to different results. The results were meaningful for the institutes using similar planning strategy and same type of TPS, so that the planners (especially the junior planners) can benefit from the planning strategy and the corresponding average performance to avoid insufficient optimization and over‐optimization as much as possible. Planners in other institutes can conduct relevant research referring to the method used in this study. (c) The continued copying after each optimization might disturb the planners, which may influence the planning time and the number of optimizations. Here we developed a script (Data S1) to incorporate the data collection and PQM scoring procedure into the Pinnacle, so that the planning strategy will be further improved, and there will be less interference to the routine clinical planning design.

## 5 CONCLUSIONS

In this study, we conducted the average performance of routine clinical lung cancer plans, and achieved similar statistical results on the plans with one or two target volumes. The longitudinal evaluation of plan quality indicated that the increasing number of optimizations was associated with significantly improved OAR sparing while only slightly affecting PTV dose coverage and homogeneity. We believe that the average performance and the planning strategy for lung cancer plans can help the planners (especially the junior planners) to design an optimal treatment plan in a more efficient way, and the average planning time can help the administrator to improve the management of clinical workflow. Furthermore, the PQM scoring procedure can help both planners and physicians to quantitively evaluate the plan quality considering various dose parameters.

## CONFLICT OF INTEREST

The authors report no conflict of interest with this study. We declare that we do not have any commercial or associative interest that represents a conflict of interest in connection with this work.

## Supporting information


**Data S1**. Pinnacle script for data collection and PQM scoring procedure
