# Supplementary material for: A longitudinal evaluation of improvements in treatment plan quality for lung cancer with volumetric modulated arc therapy
Source: J Appl Clin Med Phys. 2020 Apr 1;21(6):33–43. doi: 10.1002/acm2.12863 (PMC7324705; doi:10.1002/acm2.12863)
Supplement: Supplementary file 1 — Data S1. Pinnacle script for data collection and PQM scoring procedure [file ACM2-21-33-s001.doc]

**Main**

WindowList .PlanEval .Unrealize = "Close Window";

Store.StringAt.output = "cat << EOF > /home/path/Export_PQM/Export_Files/";

Store.At.output.AppendString = "export";

Store.At.output.AppendString = "_";

Store.At.output.AppendString = PlanInfo.MedicalRecordNumber;

Store.At.output.AppendString = "_";

Store.At.output.AppendString =DVHList.Current.TrialName;

Store.At.output.AppendString = ".txt \n";

// iteration trigger and counter

Store.FloatAt.i = 0;

Store.FloatAt.j =2;

Store.StringAt.new_line = "\n";

WindowList.DoseVolHistTabular.Create = "";

Store.StringAt.RoiName = DVHList.Current.RegionOfInterestName;

Store.At.output.AppendString = PlanInfo.LastName;

Store.At.output.AppendString = " ";

Store.At.output.AppendString = PlanInfo.MedicalRecordNumber;

Store.At.output.AppendString = " Trial: ";

Store.At.output.AppendString = DVHList.Current.TrialName;

Store.At.output.AppendString = Store.StringAt.new_line;

Store.At.output.AppendString = Store.StringAt.new_line;

// main program PQM Sum Calculation

DVHList .#"*" .BinSize =1;

Store .FloatAt .PQM_Sum=0;

Store.At.output.AppendString = "\n Structure Parameter Metric PQM ";

DVHList .ChildrenEachCurrent. #"@" .Script.ExecuteNow = "./Subroutines/Loop";

Store.At.output.AppendString = "\n PQM Sum ";

Store.At.output.AppendString=Store .FloatAt .PQM_Sum;

Store.At.output.AppendString = Store.StringAt.new_line;

// save file

SpawnCommand = Store.StringAt.output;

// clean up

KeyDependencyList.traverse_table.Destroy = "";

WindowList.DoseVolHistTabular.Unrealize = "";

Store.FreeAt.output = "";

Store.FreeAt.new_line = "";

Store.FreeAt.i = "";

Store.FreeAt.j = "";

Store.FreeAt.rows = "";

Store.FreeAt.cols = "";

Store.FreeAt.CounterString = "";

**Subroutines-Loop**

Store.StringAt.RoiName = DVHList.Current.RegionOfInterestName;

//PTV CI

IF.Store.At.RoiName.Value.Is.#"PTV".THEN.Script.ExecuteNow = "./PTV_CI.Script";

//Lung all V5

IF.Store.At.RoiName.Value.Is.#"Lung all".THEN.WindowList.DoseVolHistTabular.Create = "";

IF.Store.At.RoiName.Value.Is.#"Lung all".THEN.Script.ExecuteNow = "./Lung_All_V5.Script";

IF.Store.At.RoiName.Value.Is.#"Lung all".THEN.KeyDependencyList.traverse_table.Destroy = "";

IF.Store.At.RoiName.Value.Is.#"Lung all".THEN.WindowList.DoseVolHistTabular.Unrealize = "";

//Lung all V20

IF.Store.At.RoiName.Value.Is.#"Lung all".THEN.WindowList.DoseVolHistTabular.Create = "";

IF.Store.At.RoiName.Value.Is.#"Lung all".THEN.Script.ExecuteNow = "./Lung_All_V20.Script";

IF.Store.At.RoiName.Value.Is.#"Lung all".THEN.KeyDependencyList.traverse_table.Destroy = "";

IF.Store.At.RoiName.Value.Is.#"Lung all".THEN.WindowList.DoseVolHistTabular.Unrealize = "";

//Lung all MeanDose

IF.Store.At.RoiName.Value.Is.#"Lung all".THEN.Script.ExecuteNow = "./Lung_All_Dmean.Script";

//Heart V30

IF.Store.At.RoiName.Value.Is.#"Heart".THEN.WindowList.DoseVolHistTabular.Create = "";

IF.Store.At.RoiName.Value.Is.#"Heart".THEN.Script.ExecuteNow = "./Heart_V30.Script";

IF.Store.At.RoiName.Value.Is.#"Heart".THEN.KeyDependencyList.traverse_table.Destroy = "";

IF.Store.At.RoiName.Value.Is.#"Heart".THEN.WindowList.DoseVolHistTabular.Unrealize = "";

//Heart V40

IF.Store.At.RoiName.Value.Is.#"Heart".THEN.WindowList.DoseVolHistTabular.Create = "";

IF.Store.At.RoiName.Value.Is.#"Heart".THEN.Script.ExecuteNow = "./Heart_V40.Script";

IF.Store.At.RoiName.Value.Is.#"Heart".THEN.KeyDependencyList.traverse_table.Destroy = "";

IF.Store.At.RoiName.Value.Is.#"Heart".THEN.WindowList.DoseVolHistTabular.Unrealize = "";

//Cord MaxDose

IF.Store.At.RoiName.Value.Is.#"Cord".THEN.Script.ExecuteNow = "./Cord_Dmax.Script";

//Cord PRV MaxDose

IF.Store.At.RoiName.Value.Is.#"Cord PRV".THEN.Script.ExecuteNow = "./Cord_PRV_Dmax.Script";

**Subroutines-** **PTV_CI**

Store.FloatAt.value1=IsodoseControl .LineList .#"#0" .IsoValue;

IsodoseControl .LineList .#"#0" .IsoValue = "6000";

WindowList .IsodoseWindow .Create = "Line Details...";

IsodoseControl .LineList .#"#0" .CopyToRoiList = "Create ROI";

WindowList .IsodoseWindow .Unrealize = "Dismiss";

IsodoseControl .LineList .#"#0" .IsoValue=Store.FloatAt.value1;

WindowList .CTSim .PanelList .#"#2" .GotoPanel = "FunctionLayoutIcon2";

ViewWindowList .#"*" .CineOnOff = "0";

RoiList .Last .Name = "ROI60";

WindowList .RoiExpandWindow .Create = "ROI Expansion/Contraction...";

RoiList .#"*" .ResetRoiExpandState = "Clear All";

RoiList .Current = "PTV";

RoiList .Current .RoiExpandState = "Source";

RoiList .Current = "ROI60";

RoiList .Current .RoiExpandState = "Avoid Exterior";

RoiExpandControl .TargetRoiName = "V60PTV";

RoiExpandControl .CreateNewTarget = "1";

RoiExpandControl .UseConstantPadding = "0";

RoiExpandControl .NegXPadding = " 0";

RoiExpandControl .PosXPadding = " 0";

RoiExpandControl .NegYPadding = " 0";

RoiExpandControl .PosYPadding = " 0";

RoiExpandControl .NegZPadding = " 0";

RoiExpandControl .PosZPadding = " 0";

RoiExpandControl .Expand = "1";

RoiExpandControl .DoExpand = "Expand";

RoiList .#"*" .ResetRoiExpandState = "Clear All";

WindowList .RoiExpandWindow .Destroy = "";

WindowList .RoiDelete .Unrealize = "Dismiss";

///////////obtain volume

RoiList .Current .RecomputeStatistics = "Recompute";

RoiList .Current = "PTV";

Store .FloatAt .RoiVol1 = RoiList .Current .Volume;

RoiList .Current = "ROI60";

Store .FloatAt .RoiVol2 = RoiList .Current .Volume;

RoiList .Current = "V60PTV";

Store .FloatAt .RoiVol3 = RoiList .Current .Volume;

///////////calculate CI

Store .FloatAt .CIValue=Store .FloatAt .RoiVol3;

Store .At .CIValue.Multiply=Store .FloatAt .RoiVol3;

Store .At .CIValue.Divide=Store .FloatAt .RoiVol2;

Store .At .CIValue.Divide=Store .FloatAt .RoiVol1;

//InfoMessage=Store .FloatAt .CIValue;

Store .FloatAt .PQM_CIValue=Store .FloatAt .CIValue;

Store .FloatAt .PQM_CIValue_tmp1=0.4;

Store .FloatAt .PQM_CIValue_tmp2=1;

Store .FloatAt .PQM_CIValue_tmp3=Store .FloatAt .PQM_CIValue_tmp2;

Store .At .PQM_CIValue_tmp3 .Subtract=Store .FloatAt .PQM_CIValue_tmp1;

Store .At .PQM_CIValue .Subtract=Store .FloatAt .PQM_CIValue_tmp1;

Store .At .PQM_CIValue .Divide=Store .FloatAt .PQM_CIValue_tmp3;

Store .At .PQM_CIValue .Multiply=10;

Store.At.output.AppendString = "\n";

Store.At.output.AppendString = DVHList.Current.RegionOfInterestName;

Store.At.output.AppendString = " CI ";

Store.At.output.AppendString=Store .FloatAt .CIValue;

Store.At.output.AppendString = " ";

Store.At.output.AppendString=Store .FloatAt .PQM_CIValue;

Store .At .PQM_Sum.Add=Store .FloatAt .PQM_CIValue;

///////////delete ROI60 V60PTV

RoiList .Current = "ROI60";

CancelRoiEditing = "Delete Selected ROI (ROI60)";

RoiList .Current .Destroy = "Delete Selected ROI (ROI60)";

RoiList .Current = "V60PTV";

CancelRoiEditing = "Delete Selected ROI (V60PTV)";

RoiList .Current .Destroy = "Delete Selected ROI (V60PTV)";

**Subroutines-** **Lung_All_V5**

DVHList .Current .BinSize =500;

// move to bottom-right cell

WindowList.DoseVolHistTabular.WidgetList.Table.Table.MakePreviousCellCurrent = "";

// move back to top-left

WindowList.DoseVolHistTabular.WidgetList.Table.Table.MakeNextCellCurrent = "";

//Store.At.output.AppendString = WindowList.DoseVolHistTabular.WidgetList.Table.Table.EntryBuffer;

Store.FloatAt.Floatnum1 = WindowList.DoseVolHistTabular.WidgetList.Table.Table.EntryBuffer;

Store.At.output.AppendString = Store.StringAt.new_line;

WindowList.DoseVolHistTabular.WidgetList.Table.Table.MakeNextCellCurrent ="";

//Store.At.output.AppendString = WindowList.DoseVolHistTabular.WidgetList.Table.Table.EntryBuffer;

Store.FloatAt.Floatnum2 = WindowList.DoseVolHistTabular.WidgetList.Table.Table.EntryBuffer;

DVHList .Current .BinSize =1;

Store.FloatAt.Floatnum3=Store.FloatAt.Floatnum2;

Store.At.Floatnum3.Multiply=100;

Store.At.Floatnum3.Divide=Store.FloatAt.Floatnum1;

Store.At.Floatnum3.Multiply=100;

Store.At.Floatnum3.Round="";

Store.At.Floatnum3.Divide=100;

Store.At.output.AppendString = DVHList.Current.RegionOfInterestName;

Store.At.output.AppendString = " V5 ";

Store.At.output.AppendString=Store.FloatAt.Floatnum3;

Store.At.output.AppendString = " % ";

////////PQM

Store .FloatAt .PQM_CIValue=70;

Store .FloatAt .PQM_CIValue_tmp1=20;

Store .FloatAt .PQM_CIValue_tmp2=70;

Store .FloatAt .PQM_CIValue_tmp3=Store .FloatAt .PQM_CIValue_tmp2;

Store .At .PQM_CIValue_tmp3 .Subtract=Store .FloatAt .PQM_CIValue_tmp1;

Store .At .PQM_CIValue .Subtract=Store.FloatAt.Floatnum3;

Store .At .PQM_CIValue .Divide=Store .FloatAt .PQM_CIValue_tmp3;

Store .At .PQM_CIValue .Multiply=10;

Store.At.output.AppendString = " ";

Store.At.output.AppendString=Store .FloatAt .PQM_CIValue;

Store .At .PQM_Sum.Add=Store .FloatAt .PQM_CIValue;

Store.FreeAt.Floatnum1 = "";

Store.FreeAt.Floatnum2 = "";

Store.FreeAt.Floatnum3 = "";

**Subroutines-** **Lung_All_V20**

DVHList .Current .BinSize =2000;

// move to bottom-right cell

WindowList.DoseVolHistTabular.WidgetList.Table.Table.MakePreviousCellCurrent = "";

// move back to top-left

WindowList.DoseVolHistTabular.WidgetList.Table.Table.MakeNextCellCurrent = "";

//Store.At.output.AppendString = WindowList.DoseVolHistTabular.WidgetList.Table.Table.EntryBuffer;

Store.FloatAt.Floatnum1 = WindowList.DoseVolHistTabular.WidgetList.Table.Table.EntryBuffer;

Store.At.output.AppendString = Store.StringAt.new_line;

WindowList.DoseVolHistTabular.WidgetList.Table.Table.MakeNextCellCurrent ="";

//Store.At.output.AppendString = WindowList.DoseVolHistTabular.WidgetList.Table.Table.EntryBuffer;

Store.FloatAt.Floatnum2 = WindowList.DoseVolHistTabular.WidgetList.Table.Table.EntryBuffer;

DVHList .Current .BinSize =1;

Store.FloatAt.Floatnum3=Store.FloatAt.Floatnum2;

Store.At.Floatnum3.Multiply=100;

Store.At.Floatnum3.Divide=Store.FloatAt.Floatnum1;

Store.At.Floatnum3.Multiply=100;

Store.At.Floatnum3.Round="";

Store.At.Floatnum3.Divide=100;

Store.At.output.AppendString = DVHList.Current.RegionOfInterestName;

Store.At.output.AppendString = " V20 ";

Store.At.output.AppendString=Store.FloatAt.Floatnum3;

Store.At.output.AppendString = " % ";

////////PQM

Store .FloatAt .PQM_CIValue=28;

Store .FloatAt .PQM_CIValue_tmp1=0;

Store .FloatAt .PQM_CIValue_tmp2=28;

Store .FloatAt .PQM_CIValue_tmp3=Store .FloatAt .PQM_CIValue_tmp2;

Store .At .PQM_CIValue_tmp3 .Subtract=Store .FloatAt .PQM_CIValue_tmp1;

Store .At .PQM_CIValue .Subtract=Store.FloatAt.Floatnum3;

Store .At .PQM_CIValue .Divide=Store .FloatAt .PQM_CIValue_tmp3;

Store .At .PQM_CIValue .Multiply=10;

Store.At.output.AppendString = " ";

Store.At.output.AppendString=Store .FloatAt .PQM_CIValue;

Store .At .PQM_Sum.Add=Store .FloatAt .PQM_CIValue;

Store.FreeAt.Floatnum1 = "";

Store.FreeAt.Floatnum2 = "";

Store.FreeAt.Floatnum3 = "";

**Subroutines-** **Lung_All_Dmean**

Store.At.output.AppendString = "\n Lung all MeanDose ";

Store.At.output.AppendString =DVHList.Current.DoseMean;

Store.At.output.AppendString = " cGy ";

////////PQM

Store .FloatAt .PQM_CIValue=1700;

Store .FloatAt .PQM_CIValue_tmp1=0;

Store .FloatAt .PQM_CIValue_tmp2=1700;

Store .FloatAt .PQM_CIValue_tmp3=Store .FloatAt .PQM_CIValue_tmp2;

Store .At .PQM_CIValue_tmp3 .Subtract=Store .FloatAt .PQM_CIValue_tmp1;

Store .At .PQM_CIValue .Subtract=DVHList.Current.DoseMean;

Store .At .PQM_CIValue .Divide=Store .FloatAt .PQM_CIValue_tmp3;

Store .At .PQM_CIValue .Multiply=10;

Store.At.output.AppendString = " ";

Store.At.output.AppendString=Store .FloatAt .PQM_CIValue;

Store .At .PQM_Sum.Add=Store .FloatAt .PQM_CIValue;

**Subroutines-** **Heart_V30**

DVHList .Current .BinSize =3000;

// move to bottom-right cell

WindowList.DoseVolHistTabular.WidgetList.Table.Table.MakePreviousCellCurrent = "";

// move back to top-left

WindowList.DoseVolHistTabular.WidgetList.Table.Table.MakeNextCellCurrent = "";

//Store.At.output.AppendString = WindowList.DoseVolHistTabular.WidgetList.Table.Table.EntryBuffer;

Store.FloatAt.Floatnum1 = WindowList.DoseVolHistTabular.WidgetList.Table.Table.EntryBuffer;

Store.At.output.AppendString = Store.StringAt.new_line;

WindowList.DoseVolHistTabular.WidgetList.Table.Table.MakeNextCellCurrent ="";

//Store.At.output.AppendString = WindowList.DoseVolHistTabular.WidgetList.Table.Table.EntryBuffer;

Store.FloatAt.Floatnum2 = WindowList.DoseVolHistTabular.WidgetList.Table.Table.EntryBuffer;

DVHList .Current .BinSize =1;

Store.FloatAt.Floatnum3=Store.FloatAt.Floatnum2;

Store.At.Floatnum3.Multiply=100;

Store.At.Floatnum3.Divide=Store.FloatAt.Floatnum1;

Store.At.Floatnum3.Multiply=100;

Store.At.Floatnum3.Round="";

Store.At.Floatnum3.Divide=100;

Store.At.output.AppendString = DVHList.Current.RegionOfInterestName;

Store.At.output.AppendString = " V30 ";

Store.At.output.AppendString=Store.FloatAt.Floatnum3;

Store.At.output.AppendString = " % ";

////////PQM

Store .FloatAt .PQM_CIValue=40;

Store .FloatAt .PQM_CIValue_tmp1=0;

Store .FloatAt .PQM_CIValue_tmp2=40;

Store .FloatAt .PQM_CIValue_tmp3=Store .FloatAt .PQM_CIValue_tmp2;

Store .At .PQM_CIValue_tmp3 .Subtract=Store .FloatAt .PQM_CIValue_tmp1;

Store .At .PQM_CIValue .Subtract=Store.FloatAt.Floatnum3;

Store .At .PQM_CIValue .Divide=Store .FloatAt .PQM_CIValue_tmp3;

Store .At .PQM_CIValue .Multiply=10;

Store.At.output.AppendString = " ";

Store.At.output.AppendString=Store .FloatAt .PQM_CIValue;

Store .At .PQM_Sum.Add=Store .FloatAt .PQM_CIValue;

Store.FreeAt.Floatnum1 = "";

Store.FreeAt.Floatnum2 = "";

Store.FreeAt.Floatnum3 = "";

**Subroutines-** **Heart_V40**

DVHList .Current .BinSize =4000;

// move to bottom-right cell

WindowList.DoseVolHistTabular.WidgetList.Table.Table.MakePreviousCellCurrent = "";

// move back to top-left

WindowList.DoseVolHistTabular.WidgetList.Table.Table.MakeNextCellCurrent = "";

//Store.At.output.AppendString = WindowList.DoseVolHistTabular.WidgetList.Table.Table.EntryBuffer;

Store.FloatAt.Floatnum1 = WindowList.DoseVolHistTabular.WidgetList.Table.Table.EntryBuffer;

Store.At.output.AppendString = Store.StringAt.new_line;

WindowList.DoseVolHistTabular.WidgetList.Table.Table.MakeNextCellCurrent ="";

//Store.At.output.AppendString = WindowList.DoseVolHistTabular.WidgetList.Table.Table.EntryBuffer;

Store.FloatAt.Floatnum2 = WindowList.DoseVolHistTabular.WidgetList.Table.Table.EntryBuffer;

DVHList .Current .BinSize =1;

Store.FloatAt.Floatnum3=Store.FloatAt.Floatnum2;

Store.At.Floatnum3.Multiply=100;

Store.At.Floatnum3.Divide=Store.FloatAt.Floatnum1;

Store.At.Floatnum3.Multiply=100;

Store.At.Floatnum3.Round="";

Store.At.Floatnum3.Divide=100;

Store.At.output.AppendString = DVHList.Current.RegionOfInterestName;

Store.At.output.AppendString = " V40 ";

Store.At.output.AppendString=Store.FloatAt.Floatnum3;

Store.At.output.AppendString = " % ";

////////PQM

Store .FloatAt .PQM_CIValue=30;

Store .FloatAt .PQM_CIValue_tmp1=0;

Store .FloatAt .PQM_CIValue_tmp2=30;

Store .FloatAt .PQM_CIValue_tmp3=Store .FloatAt .PQM_CIValue_tmp2;

Store .At .PQM_CIValue_tmp3 .Subtract=Store .FloatAt .PQM_CIValue_tmp1;

Store .At .PQM_CIValue .Subtract=Store.FloatAt.Floatnum3;

Store .At .PQM_CIValue .Divide=Store .FloatAt .PQM_CIValue_tmp3;

Store .At .PQM_CIValue .Multiply=10;

Store.At.output.AppendString = " ";

Store.At.output.AppendString=Store .FloatAt .PQM_CIValue;

Store .At .PQM_Sum.Add=Store .FloatAt .PQM_CIValue;

Store.FreeAt.Floatnum1 = "";

Store.FreeAt.Floatnum2 = "";

Store.FreeAt.Floatnum3 = "";

**Subroutines-Cord_Dmax**

Store.At.output.AppendString = "\n Cord MaxDose ";

Store.At.output.AppendString =DVHList.Current.DoseMax;

Store.At.output.AppendString = " cGy ";

////////PQM

Store .FloatAt .PQM_CIValue=4000;

Store .FloatAt .PQM_CIValue_tmp1=0;

Store .FloatAt .PQM_CIValue_tmp2=4000;

Store .FloatAt .PQM_CIValue_tmp3=Store .FloatAt .PQM_CIValue_tmp2;

Store .At .PQM_CIValue_tmp3 .Subtract=Store .FloatAt .PQM_CIValue_tmp1;

Store .At .PQM_CIValue .Subtract=DVHList.Current.DoseMax;

Store .At .PQM_CIValue .Divide=Store .FloatAt .PQM_CIValue_tmp3;

Store .At .PQM_CIValue .Multiply=10;

Store.At.output.AppendString = " ";

Store.At.output.AppendString=Store .FloatAt .PQM_CIValue;

Store .At .PQM_Sum.Add=Store .FloatAt .PQM_CIValue;

**Subroutines-Cord_PRV_Dmax**

Store.At.output.AppendString = "\n Cord PRV MaxDose ";

Store.At.output.AppendString =DVHList.Current.DoseMax;

Store.At.output.AppendString = " cGy ";

////////PQM

Store .FloatAt .PQM_CIValue=4500;

Store .FloatAt .PQM_CIValue_tmp1=0;

Store .FloatAt .PQM_CIValue_tmp2=4500;

Store .FloatAt .PQM_CIValue_tmp3=Store .FloatAt .PQM_CIValue_tmp2;

Store .At .PQM_CIValue_tmp3 .Subtract=Store .FloatAt .PQM_CIValue_tmp1;

Store .At .PQM_CIValue .Subtract=DVHList.Current.DoseMax;

Store .At .PQM_CIValue .Divide=Store .FloatAt .PQM_CIValue_tmp3;

Store .At .PQM_CIValue .Multiply=10;

Store.At.output.AppendString = " ";

Store.At.output.AppendString=Store .FloatAt .PQM_CIValue;

Store .At .PQM_Sum.Add=Store .FloatAt .PQM_CIValue;
